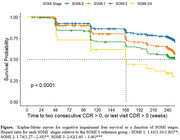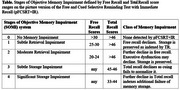# Stages of Objective Memory Impairment (SOMI) predicts clinical progression: Findings from the A4 Study

**DOI:** 10.1002/alz70857_105559

**Published:** 2025-12-25

**Authors:** Priyanka Kumari, Andrew J. Aschenbrenner, Richard B. Lipton, Ellen Grober

**Affiliations:** ^1^ Albert Einstein College of Medicine, New York, NY, USA; ^2^ Washington University in St. Louis, St. Louis, MO, USA; ^3^ Washington University School of Medicine in St. Louis, St. Louis, MO, USA; ^4^ Department of Neurology, Albert Einstein College of Medicine, Bronx, NY, USA; ^5^ Montefiore Medical Center, Bronx, NY, USA; ^6^ Albert Einstein College of Medicine, Bronx, NY, USA

## Abstract

**Background:**

Memory impairment is the hallmark cognitive deficit of Alzheimer's Disease (AD) and emerges early in the AD continuum. The Stages of Objective Memory Impairment (SOMI) is a neuropathologically validated system defined by five sequential stages in episodic memory impairment beginning in the preclinical stage of AD based on free recall (FR) and total recall (TR) scores from the Free and Cued Selective Reminding Test (FCSRT)(Table). SOMI predicted progression in two observational cohorts with similar risk profiles. Herein we report the first use of SOMI to predict progression in a clinical trial, the Anti‐Amyloid Treatment in Asymptomatic Alzheimer's(A4) study which had strict enrollment criteria.

**Method:**

Eligible participants were 1069 cognitively normal participants who had a Clinical Dementia Rating (CDR) = 0 and a positive amyloid PET scan at baseline and longitudinal data on the FCSRT Test and the CDR. Cox proportional hazards model was used to assess the association of baseline SOMI stage for clinical progression defined by the time to the first of 2 consecutive CDRs > 0. The sample was censored at 4.5 years of follow‐up.

**Results:**

Participants were on average 71.82 (SD = 4.75) years old, had 16.58 (SD = 2.80) years of education and were followed for up to 6 years (mean =  2.27, SD = 1.7 years). Most were female (59.8%), 59.5% were APOE4 positive. The distribution of SOMI stages at baseline was SOMI‐0 (483, 45.44%), SOMI‐1 (397, 37.35%) SOMI‐2 (137, 13 %), SOMI 3 (28, 3%) and SOMI 4 (17, 2%). 373 (35%) of participants were progressors. In the Cox model, using SOMI 0 as the reference the hazard ratios (HR) for progression was 1.41 (1.11–1.80, *p* = .005) for SOMI‐1, 1.74 (1.27 – 2.38, *p* <0.001) for SOMI‐2, and 2.42 (1.60 – 3.66, *p* <0.001) for SOMI 3‐4 adjusting for age, sex, education and APOE. SOMI remained an independent and significant predictor when amyloid level was added to the model. The Figure shows that cognitive impairment free survival declines in orderly fashion as SOMI stage increases (*p* < 0.0001).

**Conclusion:**

SOMI's risk profile in A4 was similar to that observed in longitudinal cohort studies despite differences in eligibility criteria.